# Immunogenicity of COVID-19 adsorbed inactivated vaccine (CoronaVac) and additional doses of mRNA BNT162b2 vaccine in immunocompromised adults compared with immunocompetent persons

**DOI:** 10.1590/S1678-9946202466024

**Published:** 2024-04-19

**Authors:** Karim Yaqub Ibrahim, Raquel Megale Moreira, Carolina Ferreira dos Santos, Tânia Mara Varejão Strabelli, Juliana de Cássia Belizário, Maria Isabel de Moraes Pinto, Ana Karolina Barreto Berselli Marinho, Juliana Marquezi Pereira, Liliane Saraiva de Mello, Mauricio Cesar Ando, Vitor Gabriel Lopes da Silva, Paula Keiko Sato, Marcos Alves de Lima, João Italo Dias França, Ana Paula Loch, Karina Takesaki Miyaji, Vanessa Infante, Alexander Roberto Precioso, Ana Marli Christovam Sartori

**Affiliations:** 1 Universidade de São Paulo, Faculdade de Medicina, Hospital das Clínicas, Divisão de Moléstias Infecciosas e Parasitarias, São Paulo, São Paulo, Brazil; 2 Universidade de São Paulo, Faculdade de Medicina, Instituto do Câncer do Estado de São Paulo, Serviço de Controle de Infecção Hospitalar, São Paulo, São Paulo, Brazil; 3 Universidade de São Paulo, Faculdade de Medicina, Hospital das Clinicas, Serviço de Transplante Renal, São Paulo, São Paulo, Brazil; 4 Universidade de São Paulo, Faculdade de Medicina, Hospital das Clinicas, Divisão de Clínica de Médica, Serviço de Hematologia, Hemoterapia e Terapia Celular, São Paulo, São Paulo, Brazil; 5 Universidade de São Paulo, Faculdade de Medicina, Hospital das Clinicas, Instituto do Coração, Subcomissão de Controle de Infecção Hospitalar, São Paulo, São Paulo, Brazil; 6 Universidade Federal de São Paulo, Departamento de Pediatria, Disciplina de Alergia, Imunologia Clínica e Reumatologia, São Paulo, São Paulo, Brazil; 7 Universidade de São Paulo, Faculdade de Medicina, Hospital das Clinicas, Departamento de Clínica Médica, Divisão de Imunologia Clínica, São Paulo, São Paulo, Brazil; 8 Universidade de São Paulo, Faculdade de Medicina, Hospital das Clinicas, Divisão de Transplante de Fígado e Órgãos do Aparelho Digestivo, São Paulo, São Paulo, Brazil; 9 Universidade de São Paulo, Faculdade de Medicina, Hospital das Clinicas, Instituto do Coração, Serviço de Pneumologia Unidade de Transplante de Pulmão, São Paulo, São Paulo, Brazil; 10 Instituto Butantan, Divisão de Ensaios Clínicos e Farmacovigilância, Laboratório Estratégico de Diagnóstico Molecular- Sorologia, São Paulo, São Paulo, Brazil; 11 Universidade Federal de São Paulo, Disciplina de Infectologia Pediátrica, Laboratório de Pesquisas, São Paulo, São Paulo, Brazil; 12 Universidade de São Paulo, Faculdade de Medicina, Laboratório de Investigação Médica-Imunologia da Divisão de Clínica de Moléstias Infecciosas e Parasitárias (LIM-48), São Paulo, São Paulo, Brazil; 13 Instituto Butantan, Divisão de Ensaios Clínicos e Farmacovigilância, Centro de Farmacovigilância, Segurança Clínica e Gestão de Risco, São Paulo, São Paulo, Brazil; 14 Universidade de São Paulo, Faculdade de Medicina, Hospital das Clínicas, Centro de Referência para Imunobiológicos Especiais, São Paulo, São Paulo, Brazil; 15 Universidade de São Paulo, Faculdade de Medicina, Departamento de Moléstias Infecciosas e Parasitarias, São Paulo, São Paulo, Brazil

**Keywords:** Vaccine immunogenicity, COVID-19 vaccines, Inactivated vaccine, BNT162 vaccine, Immunocompromised host

## Abstract

Inactivated COVID-19 vaccines data in immunocompromised individuals are scarce. This trial assessed the immunogenicity of two CoronaVac doses and additional BNT162b2 mRNA vaccine doses in immunocompromised (IC) and immunocompetent (H) individuals. Adults with solid organ transplant (SOT), hematopoietic stem cell transplant, cancer, inborn immunity errors or rheumatic diseases were included in the IC group. Immunocompetent adults were used as control group for comparison. Participants received two CoronaVac doses within a 28-day interval. IC received two additional BNT162b2 doses and H received a third BNT162b2 dose (booster). Blood samples were collected at baseline, 28 days after each dose, pre-booster and at the trial end. We used three serological tests to detect antibodies to SARS-CoV-2 nucleocapsid (N), trimeric spike (S), and receptor binding domain (RBD). Outcomes included seroconversion rates (SCR), geometric mean titers (GMT) and GMT ratio (GMTR). A total of 241 IC and 100 H adults participated in the study. After two CoronaVac doses, IC had lower SCR than H: anti-N, 33.3% vs 79%; anti-S, 33.8% vs 86%, and anti-RBD, 48.5% vs 85%, respectively. IC also showed lower GMT than H: anti-N, 2.3 vs 15.1; anti-S, 58.8 vs 213.2 BAU/mL; and anti-RBD, 22.4 vs 168.0 U/mL, respectively. After the 3^rd^ and 4^th^ BNT162b2 doses, IC had significant anti-S and anti-RBD seroconversion, but still lower than H after the 3^rd^ dose. After boosting, GMT increased in IC, but remained lower than in the H group. CoronaVac two-dose schedule immunogenicity was lower in IC than in H. BNT162b2 heterologous booster enhanced immune response in both groups.

## INTRODUCTION

The new coronavirus disease (COVID-19) pandemic has lasted more than three years and continues to pose a threat, particularly to more vulnerable groups, such as older adults and immunocompromised individuals. Vaccination has been an essential strategy to mitigate the pandemic effects. So far, 11 COVID-19 vaccines using different platforms have been recommended for emergency use by the World Health Organization (WHO)^1^. Inactivated virus vaccines are widely used worldwide, particularly in low- and middle-income countries, due to their less stringent cold chain requirements and lower costs compared to mRNA vaccines^2^. Most vaccines were licensed in a 2-dose schedule for primary immunization. Due to waning immunity, at least one booster dose, administered 4-6 months after completing the primary schedule, is currently recommended in most countries^3^. Studies show that a heterologous booster, particularly with mRNA vaccines following a primary schedule with inactivated vaccines, results in higher antibody titers and effectiveness compared with homologous booster^4,5^.

In Brazil, COVID-19 vaccination started on January 2021 with an inactivated vaccine (CoronaVac, Sinovac Biotech), two non-replicating viral vector vaccines (ChAdOx1, AstraZeneca, and Ad26.COV2.S, Janssen) and a mRNA vaccine (BNT162b2, Pfizer-BioNTech). The Brazilian Ministry of Health (MoH) provided all administered doses^6^.

Immunocompromised individuals usually have reduced immune response to vaccines compared to healthy counterparts of the same age^7^. Several phase 4 studies found lower COVID-19 vaccines immunogenicity and effectiveness in immunocompromised individuals, but most investigations were conducted in high-income countries and evaluated mRNA and viral vector vaccines^8,9^. Few studies evaluated the safety, immunogenicity, and effectiveness of inactivated COVID-19 vaccines in immunocompromised groups^10,11^.

Herein, we report the immunogenicity of CoronaVac 2-dose schedule and two additional BNT162b2 vaccine doses in immunocompromised compared with CoronaVac 2-dose schedule and one additional BNT162b2 vaccine dose in immunocompetent adults.

## MATERIAL AND METHODS

This study was conducted at the Hospital das Clinicas, FMUSP, Instituto do Coracao, FMUSP, Instituto do Cancer de Sao Paulo and Hospital Sao Paulo, UNIFESP, in Sao Paulo city, Brazil. Participants were invited from May 28 to October 6, 2021.

### Study design

A phase 4, open-label trial seeking to evaluate the safety and immunogenicity of CoronaVac 2-dose schedule in immunocompromised adults (IC) compared with immunocompetent (H) individuals, with a 12-month follow-up. The trial, started in May 2021, was adapted to be in line with MoH recommendations on COVID-19 vaccination, as follows: in September 2021, MoH recommended a third dose, administered ≥28 days after the primary schedule, for all immunocompromised individuals; in November 2021, the third vaccine dose, administered ≥4 months after the primary schedule, was extended to all adults ≥18 years; and in December 2021, a fourth dose, administered ≥4 months after the third, was recommended for the immunocompromised. According to MoH recommendations, any COVID-19 vaccine could be administered as additional doses (3^rd^ and 4^th^), regardless of which vaccine was used for the first two doses. In this study, we used mRNA BNT162b2 vaccine as additional doses.

### Study population and inclusion/exclusion criteria

Immunocompromised adults between 18 and 59 years old with SOT (liver, kidney, lung, and heart transplant), at least 30 days after transplantation and on immunosuppression; hematopoietic stem cell transplantation (HSCT), at least 30 days after autologous and 100 days after allogenic transplant, on immunosuppression or not; solid and hematological malignancies, under chemotherapy or radiotherapy or surgery within the last 6 months; inborn immunity errors with defects in antibody production; rheumatic immune-mediated diseases; end-stage chronic kidney or liver disease waiting for transplantation were invited to participate. We also included a comparison group of immunocompetent (healthy) individuals of the same age. Participants in the latter group were required to not have any known immunocompromising condition nor be undergoing immunosuppressive therapy.

Exclusion criteria included history of anaphylactic reaction to any vaccine components, previous vaccination with any COVID-19 vaccine, any vaccination within the last two weeks, any other immunocompromising condition (e.g., HIV infection, acute febrile illness or COVID-19 symptoms at enrolment), alcohol or drug addiction.

We opted for using a convenience sample, thus no sample size was calculated.

### COVID-19 vaccination

All participants received two doses of CoronaVac at a 28-day interval, according to the manufacturer’s recommendations.

**Figure 1:**
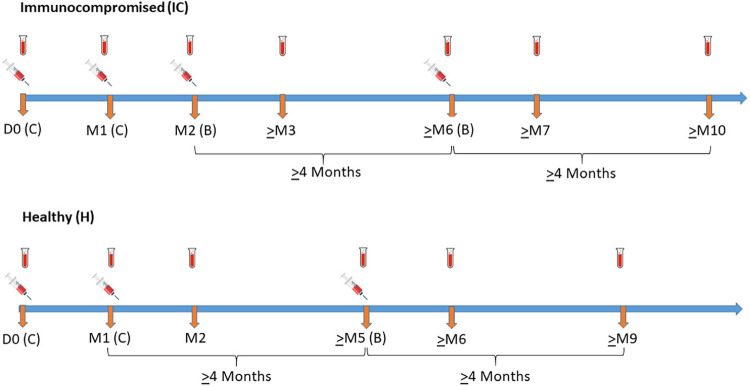
COVID-19 vaccination schedule and time of blood drawn for serological tests in immunocompromised and immunocompetent participants during the study period. C = CoronaVac; B = BNT162b2; D0 = Baseline; M = Month. Vaccination schedules: both groups received two CoronaVac (C) doses within a 28-day interval (D0 and M1). The immunocompromised (IC) group received a 3rd BNT162b2 (B) dose 28 days or more after the 2nd dose (M2) and a 4th BNT162b2 dose 4 months or more (≥M6) after the 3rd dose. The immunocompetent (H) group received a 3rd BNT162b2 dose 4 months or more after the 2nd vaccine dose (≥M5). Blood for serological tests was drawn at baseline (D0) and 28 days after each CoronaVac doses (M1 and M2) for both groups. IC also collected blood samples 28 days after the 3rd BNT162b2 dose (≥M3), before the 4th dose (≥M6), 28 days after the 4th dose (≥M7) and at trial end (≥M10). H collected blood samples before the 3rd dose (≥M5), 28 days after the 3rd BNT162b2 dose (≥M6), and at trial end (≥M9).

Immunocompromised individuals received a third vaccine dose ≥28 days after the second CoronaVac dose and a fourth dose ≥4 months after the third. Immunocompetent participants received one additional dose ≥4 months after the second CoronaVac dose (Figure 1). We analyzed only participants who received BNT162b2 vaccine as the additional doses. The primary vaccination schedule was defined as three doses (two CoronaVac plus one BNT162b2) for the IC group and two CoronaVac doses for the H group. The fourth dose for the immunocompromised and the third dose for the immunocompetent were considered boosters.

CoronaVac doses were supplied by Instituto Butantan. BNT162b2 doses were obtained from the COVID-19 National Immunization Program, supplied by MoH. Description of the vaccine batches used is given in the Supplementary Files. In case of COVID-19 infection, the subsequent vaccination was delayed by four weeks, according to MoH recommendations.

### Study outcomes and definitions

Vaccine immunogenicity was assessed by seroconversion rates (SCR), geometric mean titers (GMT) and geometric mean titers ratio (GMTR) between post-vaccination and baseline values, 28 days after each dose, pre-booster and at trial end (Figure 1).

Seropositivity was defined for each serological test according to manufacturer’s instructions (described in Laboratory procedures, below). Seroconversion was defined as seropositivity after vaccination in a pre-vaccination seronegative person or 4-fold increase in antibody titers between pre- and post-vaccination.

### Study procedures

Demographic and clinical data of the participants were collected at baseline. Data on previous SARS-CoV-2 infection was not collected. Blood samples for anti-SARS-CoV-2 serological testing were collected at baseline (Day 0), 28 days after each vaccine dose, before the booster and at trial end, which was four months after the booster (Figure 1).

### Laboratory procedures

After blood drawn, the samples were kept at room temperature (for up to three hours) until transport to the Laboratory of Medical Investigation in Immunology (LIM-48, HC-FMUSP). After centrifugation at 1,500 g for 10 min, serum samples were aliquoted and stored at −20 °C until transport to the Strategic Laboratory of Molecular Diagnosis/Serology, Instituto Butantan, for analysis. Anti-SARS-CoV antibodies were detected using three serological tests:

- An electro-chemiluminescent immunoassay (ECLIA) for qualitative detection of antibodies targeting SARS-CoV2 nucleocapsid (N) protein (Elecsys^®^ anti-SARS-CoV-2, by Roche Diagnostics) that evaluates adaptive immune response to previous infection or to CoronaVac vaccination but not to BNT162b2 vaccine, since the latter does not contain the N protein. Seropositivity was defined as COI (cut-off index) ≥1.0 antibody units (UA)/mL.
- A chemiluminescent immunoassay (CLIA) for quantitative determination of IgG antibodies targeting the SARS-CoV-2 trimeric spike (S) protein (LIAISON^®^ SARS-CoV-2 TrimericS IgG, DiaSorin). Seropositivity was defined as IgG ≥33.8 binding antibody units (BAU)/mL.
- An electro-chemiluminescent immunoassay (ECLIA) for quantitative determination of antibodies targeting the SARS-CoV-2 spike protein receptor-binding domain (RBD) (Elecsys^®^ Anti-SARS-CoV-2 S, by Roche Diagnostics). Seropositivity was defined as anti-RBD test ≥0.8 U/mL.

### Vaccines batches

The primary vaccination schedule used the following CoronaVac batches: 20200412, 202009004, 210441A, 210473, 210218, C202106107, 210223, 210325, 210320A, 2028840, 210481, 210413, 210441A, 210473, and 210476.

As third dose, we used the following BNT162b2 batches: FG3533, FG3530, FG3531, FG3524, FG3530, FG3529, FG3531, FF8846, FF5108, FK9412, FH8026, FL3207, FK8911, FK8917, FJ4187, FF8842, FM3355, FH4751, FM2952, FM2953, FM2967, FG3535, FM7380, and FN9606.

### Statistical analysis

Descriptive approaches by study group were employed to meet the protocol objectives. Continuous variables were summarized using descriptive statistics—non-missing participants number (n), median (Q2) and quartiles (Q1, Q3). Frequency (n) and percentages (%) for categorical variables, based on non-missing participants, were reported for each study group. Immunogenicity data, for each time point by study group, were presented as GMT, GMT ratio between baseline and post-vaccination values (GMTR), seroconversion rates (SCR) and seropositivity rates (SPR). GMT and GMTR data are quantitative, whereas seroconversion and seropositivity data are qualitative. GMT and GMTR were calculated as the anti-logarithm of the log-transformed titer mean.

The 95% confidence interval (95%CI) were calculated as the anti-logarithm transformation of the upper and lower limits of a two-sided CI for the log-transformed titers mean. Seroconversion and seropositivity percentages were calculated for each study group along with its respective 95%CI using Clopper-Pearson’s method. Titers below the lowest quantitation limit were set to half that limit. If a titer was greater or equal to the assay’s upper limit, it was set to that limit. Comparisons between the IC and H groups, at each time point, were performed by Mann-Whitney’s test for continuous variables and Fisher’s exact test for categorical variables. At each time point, all vaccinated participants with available blood sample were included in the analysis.

### Ethical approval statement

The original protocol and all changes made during the trial were approved by the Ethics Committees of the participating institutions and the National Research Ethics Committee (CONEP, Comissao Nacional de Etica em Pesquisa, CAAE Nº 87498318.0.0000.0068). The protocol was registered in the Brazilian Registry of Clinical Trials (REBEC, RBR-9ksh5f4). All participants provided written informed consent before enrollment and at each protocol change. Participant identification remained confidential throughout the study and analyses.

## RESULTS

From May 28 to October 6, 2021, 341 participants were included in the study: 241 immunocompromised (114 SOT recipients, 30 HSCT recipients, 27 cancer patients, 44 patients with IEI, 21 individuals with rheumatic diseases and five with end-stage chronic diseases pre-transplantation) and 100 immunocompetent participants. There were 129 (53.5%) women in the immunocompromised group (IC) and 48 (48%) women in the immunocompetent group (H). Median age was 36 (interquartile range, IQR 26.0-50.0) and 37 years (IQR, 31.0-44.0), respectively. Demographic profile of immunocompromised and immunocompetent participants were similar, except for schooling years (Table 1).

**Table 1:** Demographic profile of participants in the study of immunogenicity of COVID-19 adsorbed inactivated vaccine (CoronaVac) and additional mRNA BNT162b2 vaccine doses in immunocompromised adults compared with immunocompetent persons. Sao Paulo, Brazil, 2021-2022.

	Immunocompromised (IC) (n=241)		Immunocompetent (H) (n=100)		p-value^**1**^
Age (years)					
Median (Q_1_-Q_3_)	36.0	(26.0 – 50.0)	37.0	(31.0 – 44.0)	
**Gender, n (%)**					0.405
Female	129	(53.5)	48	(48.0)	
Male	112	(46.5)	52	(52.0)	
**Ethnicity, n (%)**					0.633
White	135	(56.0)	59	(59.0)	
Non-white	106	(44.0)	41	(41.0)	
**Years of study**					**<0.001**
Median (Q_1_-Q_3_)	11	(9 - 12)	15	(11 - 16)	

**Figure 2:**
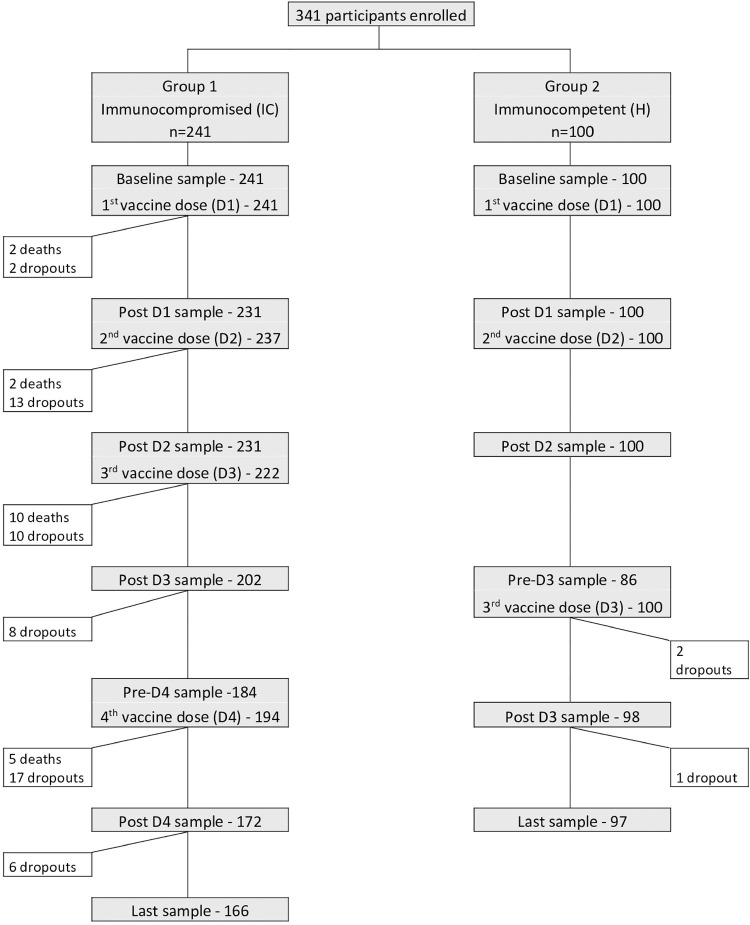
Number of participants enrolled, vaccinated and who collected blood samples for serological tests; deaths and dropouts in both immunocompromised and immunocompetent groups at each time point.

All 341 participants collected blood samples at baseline and received the 1^st^ CoronaVac dose; 237 IC and all 100 H participants received the 2^nd^ CoronaVac dose; 222 immunocompromised and 100 immunocompetent participants received the 3^rd^ BNT162b2 mRNA vaccine dose; and 194 immunocompromised participants received the 4^th^ BNT162b2 mRNA vaccine dose. Mean interval between the 1^st^ and 2^nd^ doses was 28 days (Q1 = 28, Q3 = 28) in both IC and H groups; and the mean interval between the 2^nd^ and 3^rd^ doses was 76 days (Q1 = 62, Q3 = 98) in the IC group. Figure 2 presents the number of participants (IC and H) that were vaccinated and collected blood samples at each time point. We registered 19 deaths among the IC (four due to COVID and all others related to the underlying condition) and 56 dropouts in IC and 3 dropouts in H. A total of 263 participants completed the study (Figure 2).

At baseline, the immunocompromised had greater seropositivity rates in all three serological tests: 33.2% of IC and 22% of H (p=0.051) were seropositive to anti-N (Table 2A); 31.5% of IC and 18% of H (p=0.011) were seropositive to anti-S (Table 2B); and 45.2% of IC and 22% of H (p<0.001) were seropositive to anti-RBD (Table 2C), suggesting higher rates of previous SARS-COV-2 infection among the IC.

**Table 2A:** Anti-SARS-CoV nucleocapsid (N) seropositivity rates (SPR), seroconversion rates (SCR), antibody geometric mean titers (GMT), and GMT ratios (GMTR) in the immunocompromised (IC) and immunocompetent (H) groups at baseline, 28 days after each vaccine dose, pre-booster (3rd dose in immunocompetent and 4th immunocompromised) and at trial end (4 months after booster).

	Immunocompromised (IC)			Immunocompetent (H)			p-value
	
	n	value	95%CI	n	value	95%CI	
**SPR,%**							
Basal	241	33.2	(27.3 – 39.5)	100	22.0	(14.3 – 31.4)	0.051
post-V1	231	38.5	(32.2 – 45.1)	100	27.0	(18.6 – 36.8)	**0.046**
post-V2	231	51.9	(45.3 – 58.5)	100	91.0	(83.6 – 95.8)	**<0.001**
pre-V3	.	.	.	86	77.9	(67.7 – 86.1)	.
post-V3	202	58.4	(51.3 – 65.3)	98	78.6	(69.1 – 86.2)	**<0.001^1^**
pre-V4	184	67.9	(60.7 – 74.6)	.	.	.	0.113^2^
post-V4	172	67.4	(59.9 – 74.4)	.	.	.	0.068^3^
End	166	75.3	(68.0 – 81.7)	97	86.6	(78.2 – 92.7)	**0.039**
**SCR,%**							
post-V1	231	16.9	(12.3 – 22.3)	100	13.0	(7.1 – 21.2)	0.415
post-V2	231	33.3	(27.3 – 39.8)	100	79.0	(69.7 – 86.5)	**<0.001**
pre-V3	.	.	.	86	65.1	(54.1 – 75.1)	.
post-V3	202	41.6	(34.7 – 48.7)	98	65.3	(55.0 – 74.6)	**<0.001^1^**
pre-V4	184	52.7	(45.2 – 60.1)	.	.	.	0.065^2^
post-V4	172	51.7	(44.0 – 59.4)	.	.	.	**0.041^3^**
End	166	62.0	(54.2 – 69.5)	97	73.2	(63.2 – 81.7)	0.079
**GMT**							
Basal	241	0.5	(0.4 – 0.8)	100	0.3	(0.2 – 0.5)	**<0.001**
post-V1	231	1.0	(0.7 – 1.5)	100	0.7	(0.4 – 1.2)	0.820
post-V2	231	2.3	(1.5 – 3.4)	100	15.1	(10.3 – 22.3)	**<0.00**1
pre-V3	.	.	.	86	7.2	(4.5 – 11.5)	.
post-V3	202	2.8	(1.9 – 4.2)	98	10.0	(6.3 – 16.0)	**<0.001^1^**
pre-V4	184	6.5	(4.3 – 9.8)	.	.	.	0.740^2^
post-V4	172	7.0	(4.6 – 10.7)	.	.	.	0.265^3^
End	166	10.6	(7.0 – 15.9)	97	33.6	(21.5 – 52.5)	**<0.001**
**GMTR**							
post-V1	231	1.8	(1.5 – 2.1)	100	2.2	(1.8 – 2.7)	**<0.001**
post-V2	231	4.2	(3.2 – 5.5)	100	49.4	(32.0 – 76.3)	**<0.001**
pre-V3	.	.	.	86	25.9	(15.4 – 43.5)	.
post-V3	202	5.7	(4.1 – 7.8)	98	34.4	(19.9 – 59.3)	**<0.001^1^**
pre-V4	184	13.6	(9.0 – 20.6)	.	.	.	**0.028^2^**
post-V4	172	13.1	(8.4 – 20.5)	.	.	.	**0.004^3^**
End	166	22.6	(14.8 – 34.7)	97	113.3	(60.5 – 212.3)	**<0.001**

SPR = seropositivity rate (seropositivity was defined as COI (cut-off index) ≥1.0); SCR = seroconversion rate (negative pre-vaccination titer and a positive post-vaccination titer or positive pre-vaccination titer and at least a four-fold increase in post-vaccination measure); GMT = geometric mean titers; GMTR = geometric mean titer ratio (ratio between post-dose administration and baseline GMT); Basal = sample collected before administering the 1^st^ dose; post-V1 = 28 days after 1^st^ dose; post-V2 = 28 days after 2^nd^ dose; post-V3 = 28 days after 3^rd^ dose; post-V4 = 28 days after 4^th^ dose; NP = not possible to calculate; *Tests used to compare immunocompromised and immunocompetent groups (Mann-Whitney’s test for GMT and GMTR and Fisher’s exact test for SCR and SPR); ^1^comparison between post-V3 in immunocompromised and post-V2 in immunocompetent (H); ^2^comparison between pre-V4 in immunocompromised and pre-V3 in immunocompetent (H); ^3^comparison between post-V4 in immunocompromised and post-V3 in immunocompetent (H).

**Table 2B:** Anti-SARS-CoV spike (S) seropositivity rates (SPR), seroconversion rates (SCR), antibody geometric mean titers (GMT), and GMT ratios (GMTR) in the immunocompromised (IC) and immunocompetent (H) groups at baseline, 28 days after each vaccine dose, pre-booster (3rd dose in immunocompetent and 4th immunocompromised) and at trial end (4 months after booster).

	Immunocompromised (IC)			Immunocompetent (H)			p-value
	
	n	value	95%CI	n	value	95%CI	
**SPR,%**							
Basal	241	31.5	(25.7 – 37.8)	100	18.0	(11.0 – 26.9)	**0.011**
post-V1	231	45.9	(39.3 – 52.5)	100	42.0	(32.2 – 52.3)	0.548
post-V2	231	59.3	(52.7 – 65.7)	100	96.0	(90.1 – 98.9)	**<0.001**
pre-V3	.	.	.	86	55.8	(44.7 – 66.5)	.
post-V3	202	81.7	(75.6 – 86.8)	98	100.0	(96.3 – 100.0)	**<0.001^1^**
pre-V4	184	85.9	(80.0 – 90.6)	.	.	.	**<0.001^2^**
post-V4	172	89.0	(83.3 – 93.2)	.	.	.	**<0.001^3^**
End	166	88.0	(82.0 – 92.5)	97	100.0	(96.3 – 100.0)	**<0.001**
**SCR,%**							
post-V1	231	19.0	(14.2 – 24.7)	100	32.0	(23.0 – 42.1)	**0.015**
post-V2	231	33.8	(27.7 – 40.3)	100	86.0	(77.6 – 92.1)	**<0.001**
pre-V3	.	.	.	86	43.0	(32.4 – 54.2)	.
post-V3	202	71.3	(64.5 – 77.4)	98	99.0	(94.4 – 100.0)	**0.006^1^**
pre-V4	184	72.8	(65.8 – 79.1)	.	.	.	**<0.001^2^**
post-V4	172	79.1	(72.2 – 84.9)	.	.	.	**<0.001^3^**
End	166	81.3	(74.6 – 86.9)	97	92.8	(85.7 – 97.0)	**0.011**
**GMT**							
Basal	241	14.5	(10.9 – 19.3)	100	6.0	(4.3 – 8.3)	**<0.001**
post-V1	231	28.8	(21.1 – 39.3)	100	39.8	(28.8 – 54.9)	0.081
post-V2	231	58.8	(43.6 – 79.3)	100	213.2	(173.4 – 262.2)	**<0.001**
pre-V3	.	.	.	86	76.0	(51.9 – 111.4)	.
post-V3	202	393.2	(282.1 – 548.0)	98	1500.7	(1376.3 – 1636.3)	**<0.001^1^**
pre-V4	184	401.8	(290.7 – 555.4)	.	.	.	**<0.001^2^**
post-V4	172	731.8	(542.5 – 987.2)	.	.	.	0.386^3^
End	166	730.0	(538.3 – 990.0)	97	1405.2	(1241.9 – 1590.1)	0.419
**GMTR**							
post-V1	231	2.0	(1.7 – 2.3)	100	6.7	(5.5 – 8.2)	**<0.001**
post-V2	231	4.1	(3.2 – 5.1)	100	35.8	(26.2 – 48.8)	**<0.001**
pre-V3	.	.	.	86	13.2	(8.5 – 20.4)	.
post-V3	202	30.9	(21.8 – 43.8)	98	255.8	(183.8 – 356.1)	0.695^1^
pre-V4	184	30.9	(21.5 – 44.6)	.	.	.	**0.007^2^**
post-V4	172	54.5	(37.4 – 79.4)	.	.	.	**<0.001^3^**
End	166	62.6	(43.4 – 90.2)	97	237.4	(160.6 – 350.8)	**<0.001**

SPR = seropositivity rate (seropositivity was defined as anti-S ≥33.8 binding antibody units (BAU)/mL); SCR = seroconversion rate (negative pre-vaccination titer and a positive post-vaccination titer or positive pre-vaccination titer and at least a four-fold increase in post-vaccination measure); GMT = geometric mean titers; GMTR = geometric mean titer ratio (ratio between post-dose administration and baseline GMT); Basal = sample collected before administering the 1^st^ dose; post-V1 = 28 days after 1^st^ dose; post-V2 = 28 days after 2^nd^ dose; post-V3 = 28 days after 3^rd^ dose; post-V4 = 28 days after 4^th^ dose; NP = not possible to calculate; *Tests used to compare immunocompromised and immunocompetent groups (Mann-Whitney’s test for GMT and GMTR and Fisher’s exact test for SCR and SPR); ^1^comparison between post-V3 in immunocompromised and post-V2 in immunocompetent (H); ^2^comparison between pre-V4 in immunocompromised and pre-V3 in immunocompetent (H); ^3^comparison between post-V4 in immunocompromised and post-V3 in immunocompetent (H).

**Table 2C:** Anti-SARS-CoV spike receptor binding domain (RBD) seropositivity rates (SPR), seroconversion rates (SCR), antibody geometric mean titers (GMT), and GMT ratios (GMTR) in the immunocompromised (IC) and immunocompetent (H) groups at baseline, 28 days after each vaccine dose, pre-booster (3rd dose in immunocompetent and 4th immunocompromised) and at trial end (4 months after booster).

	Immunocompromised (IC)			Immunocompetent (H)			p-value
	
	n	value	95%CI	n	value	95%CI	
**SPR,%**							
Basal	241	45.2	(38.8 – 51.7)	100	22.0	(14.3 – 31.4)	**<0.001**
post-V1	231	60.6	(54.0 – 67.0)	100	90.0	(82.4 – 95.1)	**<0.001**
post-V2	231	78.4	(72.5 – 83.5)	100	100.0	(96.4 – 100.0)	**<0.001**
pre-V3	.	.	.	86	100.0	(95.8 – 100.0)	.
post-V3	202	92.1	(87.5 – 95.4)	98	100.0	(96.3 – 100.0)	**0.002^1^**
pre-V4	184	94.0	(89.6 – 97.0)	.	.	.	**0.019^2^**
post-V4	172	96.5	(92.6 – 98.7)	.	.	.	**0.090^3^**
End	166	95.2	(90.7 – 97.9)	97	100.0	(96.3 – 100.0)	0.028
**SCR,%**							
post-V1	231	25.5	(20.0 – 31.7)	100	75.0	(65.3 – 83.1)	**<0.001**
post-V2	231	48.5	(41.9 – 55.1)	100	85.0	(76.5 – 91.4)	**<0.001**
pre-V3	.	.	.	86	84.9	(75.5 – 91.7)	.
post-V3	202	68.3	(61.4 – 74.7)	98	85.7	(77.2 – 92.0)	**0.002^1^**
pre-V4	184	70.7	(63.5 – 77.1)	.	.	.	**0.015^2^**
post-V4	172	72.7	(65.4 – 79.2)	.	.	.	**0.015^3^**
End	166	74.1	(66.7 – 80.6)	97	85.6	(77.0 – 91.9)	**0.031**
**GMT**							
Basal	241	2.6	(1.8 – 3.8)	100	0.7	(0.5 – 1.2)	**<0.001**
post-V1	231	6.6	(4.4 – 9.8)	100	9.1	(6.0 – 14.0)	0.267
post-V2	231	22.4	(15.6 – 32.2)	100	168.0	(143.8 – 196.3)	**<0.001**
pre-V3	.	.	.	86	79.3	(61.4 – 102.6)	.
post-V3	202	97.4	(72.7 – 130.6)	98	250.0	(250.0 – 250.0)	**0.008^1^**
pre-V4	184	116.7	(88.5 – 153.8)	.	.	.	**<0.001^2^**
post-V4	172	157.0	(125.3–196.7)	.	.	.	**<0.001^3^**
End	166	151.0	(117.3 – 194.3)	97	250.0	(250.0 – 250.0)	**<0.001**
**GMTR**							
post-V1	231	2.5	(2.0 – 3.1)	100	12.2	(9.0 – 16.6)	**<0.001**
post-V2	231	8.6	(6.2 – 12.0)	100	224.1	(137.8 – 364.3)	**<0.001**
pre-V3	.	.	.	86	111.4	(67.7 – 183.4)	.
post-V3	202	41.7	(27.6 – 63.1)	98	340.8	(205.7 – 564.5)	**0.001^1^**
pre-V4	184	50.5	(32.6 – 78.3)	.	.	.	0.263^2^
post-V4	172	60.4	(38.5 – 94.7)	.	.	.	**<0.001^3^**
End	166	66.1	(41.9 – 104.3)	97	338.8	(203.5 – 564.2)	**<0.001**

SPR = seropositivity rate (seropositivity was defined as anti-RBD test ≥0.8 U/mL); SCR = seroconversion rate (negative pre-vaccination titer and a positive post-vaccination titer or positive pre-vaccination titer and at least a four-fold increase in post-vaccination measure); GMT = geometric mean titers; GMTR = geometric mean titer ratio (ratio between post-dose administration and baseline GMT); Basal = sample collected before administering the 1^st^ dose; post-V1 = 28 days after 1^st^ dose; post-V2 = 28 days after 2^nd^ dose; post-V3 = 28 days after 3^rd^ dose; post-V4 = 28 days after 4^th^ dose; NP = not possible to calculate; *Tests used to compare immunocompromised and immunocompetent groups (Mann-Whitney’s test for GMT and GMTR and Fisher’s exact test for SCR and SPR); ^1^comparison between post-V3 in immunocompromised and post-V2 in immunocompetent (H); ^2^comparison between pre-V4 in immunocompromised and pre-V3 in immunocompetent (H); ^3^comparison between post-V4 in immunocompromised and post-V3 in immunocompetent (H).

### Anti-nucleocapsid (N) immune response

Anti-N seroconversion rates (SCR) 28 days after the 1^st^ CoronaVac dose did not differ statistically between groups: 16.9% of IC and 13% of H seroconverted (p=0.415) (Table 2A). After the 2^nd^ CoronaVac dose, IC (33.3%) showed lower anti-N SCR compared with immunocompetent (79%) and this difference was statistically significant (p<0.001). After the 3^rd^ BNT162b2 dose, anti-N SCR increased to 41.6% in the IC group, but was still lower than in the H group (79.0%) after two doses (p<0.001). Before the booster (pre-V4 in IC and pre-V3 in H) anti-N SCR increased in the IC group to 52.7% against a mild decrease in H (65.1%), without statistically significant differences between groups (p=0.065). Anti-N SCR 28 days after the booster showed no differences: 51.7% in IC and 65.3% in H (p= 0.041). Once the study ended (4 months after the booster dose), anti-N SCR levels increased in both IC (to 62.0%) and H (to 73.2%), without statistically significant difference between groups (p= 0.079).

**Figure 3:**
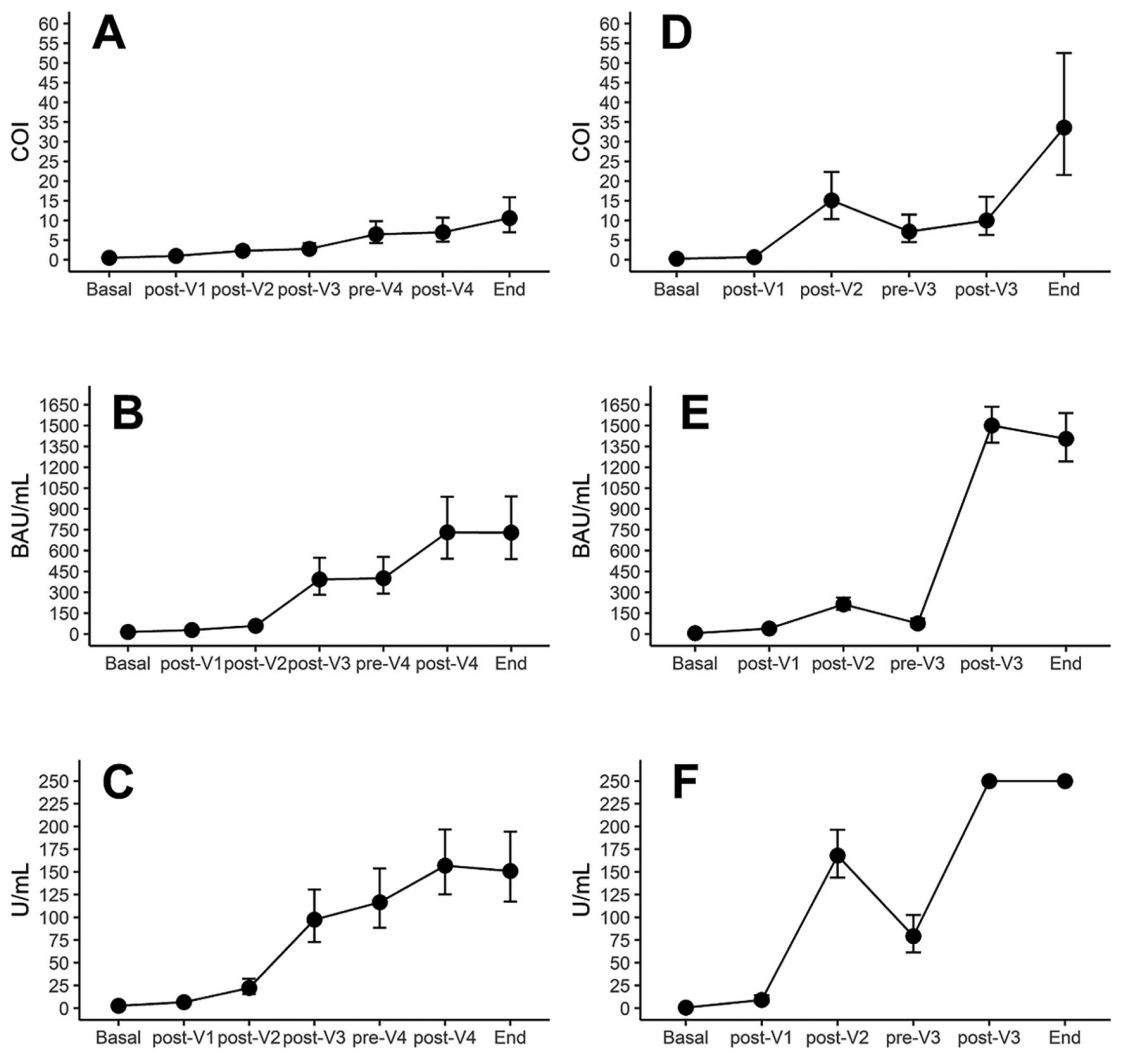
Anti-N, anti-S, and anti-RBD geometric mean titers (GMT) in the immunocompromised and immunocompetent groups at each time point. A) anti-N GMT measured in COI from baseline to months after vaccination in the immunocompromised group; B) anti-S GMT measured in BAU/mL from baseline to months after vaccination in the immunocompromised group; C) anti-RBD GMT measured in U/mL from baseline to months after vaccination in the immunocompromised group; D) anti-N GMT measured in COI from baseline to months after vaccination the immunocompetent group; E) anti-S GMT measured in BAU/mL from baseline to months after vaccination in the immunocompetent group; F) anti-RBD GMT measured in U/mL from baseline to months after vaccination in the immunocompetent group.

At baseline, anti-N GMT was higher in immunocompromised (0.5) than in immunocompetent (0.3) participants (p<0.001) (Table 2A). After the first CoronaVac dose, we observed no difference in anti-N GMT between the IC (1.0) and H (0.7) groups (p=0.82). After the 2^nd^ CoronaVac dose, anti-N GMT was significantly lower in IC (2.3) than in H (15.1) (p<0.001). As expected, the 3^rd^ BNT162b2 dose did not significantly increase anti-N GMT in the IC group (from 2.3 to 2.8), which remained lower than in H after the second dose (15.1) (p<0.001) (Table 2A, Figures 3A and 3D). Before the booster (pre-V4 in IC and pre-V3 in H), anti-N GMT increased in the IC group (to 6.5) and reduced in the H (to 7.2), but without statistically significant difference between groups (p=0.740). We observed no difference in anti-N GMT between groups 28 days after boosting: 7.0 in IC and 10.0 in H (p=0.265). Four months after the boost dose, anti-N GMT increased in both IC (to 10.6) and H (to 33.6), and the difference was statistically significant (p<0.001) (Table 2A, Figures 3A and 3D).

GMTR showed lower anti-N titers increases in immunocompromised than in H. After the 1^st^ CoronaVac dose, anti-N titers increased 1.8 and 2.2, respectively, in relation to baseline (p<0.001). Following the 2^nd^ CoronaVac dose, anti-N GMT increased 4.2 times in IC and 49.4 times in H (p<0.001). As expected, the 3^rd^ BNT162b2 dose did not significantly increase anti-N GMTR in the IC group (from 4.2 to 5.7) and anti-N GMTR remained lower than after the second dose in H (49.4) (p<0.001). Before the booster (pre-V4 in IC and pre-V3 in H), anti-N GMTR increased in IC (to 13.6) and reduced in H (to 25.9) and the difference between the groups persisted (p=0.028). Anti-N GMTR levels 28 days after boosting showed no difference in IC (13.1), whereas the H group presented a mild increase to 34.4 (p=0.004). At the end of the study, anti-N GMTR increased in both groups, to 22.6 in IC and to 113.3 in H, and the difference was statistically significant (p<0.001) (Table 2A).

### Anti-Spike (S) immune response

Anti-S seroconversion was significantly lower in IC after both 1^st^ and 2^nd^ CoronaVac doses (Table 2B). The 1^st^ dose elicited anti-S seroconversion in 19% of IC and 32% of H (p=0.015). The 2^nd^ dose increased anti-S SCR to 33.8% in IC and 86% in H (p<0.001). After the 3^rd^ BNT162b2 dose, anti-S SCR increased to 71.3% in the IC group, still a lower percentage than in the H group after two doses (86%) (p=0.006). Before the booster (pre-V4 in IC and pre-V3 in H), we observed a mild increase in anti-S SCR in IC, to 72.8%, against a decrease of anti-S SCR in H, to 43.0% (p<0.001). Anti-S SCR 28 days after the booster dose increased in both groups, but SCR was still lower in IC (79.1%) than in H (99.0%) (p<0.001). Four months after the booster dose, anti-S SCR levels increased in IC to 81.3% and a mildly decreased in H to 92.8%, but the difference between groups persisted (p=0.011).

At baseline, anti-S GMT was higher in immunocompromised (14.5 BAU/mL) than in immunocompetent (6.0 BAU/mL) participants (p<0.001). One month after the 1^st^ CoronaVac dose, anti-S GMT was lower in the IC group (28.8 BAU/mL), but not statistically different from H (39.8 BAU/mL) (p=0.081). After the 2^nd^ CoronaVac dose, anti-S GMT was statistically lower in IC (58.8 BAU/mL) than in H (213.2 BAU/mL) (p<0.001). The 3^rd^ BNT162b2 dose significantly increased anti-S GMT in IC (393.2), becoming significantly higher than anti-S GMT in H after the 2^nd^ dose (213.2) (p<0.001) (Table 2B, Figures 3B and 3E). Before the booster (pre-V4 in IC and pre-V3 in H), anti-S GMT mildly increased in IC (to 401.8) and decreased in H (to 76.0), and anti-S GMT was significantly higher in IC (p<0.001). Anti-S GMT 28 days after the booster dose increased in both IC (to 731.8) and H (to 1,500.7), without statistically significant difference between groups (p=0.386). At the end of the study, anti-S GMT levels remained high in both IC (730.0) and H (1,405.2), without statistically significant difference between groups (p=0.419) (Table 2B, Figures 3B and 3E).

GMTR analysis showed that anti-S GMT increased 2.0 times in IC and 6.7 times in H after the 1^st^ CoronaVac dose (p<0.001). After the 2^nd^ CoronaVac dose, anti-S GMT increased 4.1 and 35.8 times in IC and H, respectively (p<0.001). The 3^rd^ BNT162b2 dose significantly increased anti-S GMTR in IC to 30.9, almost reaching anti-S GMTR values in H after the 2^nd^ dose (35.8), but the difference was not statistically significant (p= 0.695) (Table 2B). Before the booster (pre-V4 in IC and pre-V3 in H), anti-S GMTR showed no changes in IC (30.9) but was significantly higher than in H, which decrease (to 13.2) (p=0.007). Immunocompromised had a statistically significant lower increase in anti-S GMTR 28 days after booster dose (to 54.5) compared with the H group (to 255.8) (p<0.001). At the end of the study, anti-S GMTR mildly increased in IC (to 62.6) and mildly decreased in H (to 237.4), but anti-S GMTR remained significantly lower in IC (p<0.001) (Table 2B).

### Anti-Receptor Binding Domain (RBD) immune response

Anti-RBD immune responses followed a similar pattern, with a significantly weaker response in immunocompromised (Table 2C). The 1^st^ CoronaVac dose elicited anti-RBD seroconversion in 25.5% of IC and 75% of H (p<0.001). After the 2^nd^ CoronaVac dose, anti-RBD SCR increased to 48.5% in IC and 85% in H, and the difference between groups was statistically significant (p<0.001). The 3^rd^ BNT162b2 dose increased seroconversion to 68.3% in IC, but SCR remained lower than in the H group after two doses (85%) (p=0.002). Before the booster (pre-V4 in IC and pre-V3 in H), anti-RBD SCR mildly increased in IC (to 70.7%), but still lower than in the H (84.9%) (p=0.015). The booster produced no significant anti-RBD SCR change whether in IC (to 72.7%) or in H (to 85.7%) and the difference between groups remained statistically significant (p=0.015). We observed no important change in anti-RBD SCR at the end of the study: 74.1% in IC and 85.6% in H, and the difference between the groups was still significant (p=0.031).

At baseline, anti-RBD GMT was higher in the IC group (2.6 U/mL) than in the H group (0.7 U/mL) (p<0.001). After the 1^st^ CoronaVac dose, we observed no significant difference in anti-RBD GMT between the groups: 6.6 U/mL in IC and 9.1 U/mL in H (p=0.267). After the 2^nd^ CoronaVac dose, anti-RBD GMT increased in both groups, but was significantly lower in IC (22.4 U/mL) than in H (168.0 U/mL) (p<0.001). The 3^rd^ BNT162b2 dose significantly increased anti-RBD GMT in IC to 97.4; however, this value was still lower than anti-RBD GMT in H after the 2^nd^ dose (168.0) (p=0.008) (Table 2C, Figures 3C and 3F). Before the booster (pre-V4 in IC and pre-V3 in H), anti-RBD GMT increased in IC (to 116.7) and decreased in H (to 79.3), and the difference between groups was statistically significant (p<0.001). Anti-RBD GMT increased 28 days after the booster in both groups: to 157.0 in IC and to 250.0 in H, and the difference was still statistically significant (p<0.001). At the end of the study, we observed no important changes in anti-RBD GMT values, which remained lower in IC (151.0) than in H (250.0) (p<0.001) (Table 2C, Figures 3C and 3F).

After the 1^st^ CoronaVac dose, GMTR analysis showed that anti-RBD titers presented a lower increase in IC (2.5 times) than in H (12.2 times) (p<0.001). After the 2^nd^ CoronaVac dose, anti-RBD GMTR increased to 8.6 and 224.1 in IC and H, respectively (p<0.001). The 3^rd^ BNT162b2 dose increased anti-RBD GMTR in IC to 41.7; however, it was still lower than in H after the 2^nd^ dose (224.1) (p<0.001) (Table 2C). Before the booster (pre-V4 in IC and pre-V3 in H), anti-RBD GMTR mildly increased in IC (50.5) and decreased in H (111.4) (p=0.263). Anti-RBD GMTR increased in both groups 28 days after the booster: to 60.4 in IC and to 340.8 in H, and the difference between groups was statistically significant (p<0.001). At the end of the study, anti-RBD GMTR increased to 66.1 in the IC group, but it was still significantly lower than in H (338.8) (p<0.001).

### COVID-19 episodes throughout the study period

During the trial, we diagnosed 96 COVID-19 episodes confirmed by a positive SARS-CoV-2 PCR or antigen test: 54 episodes in immunocompromised and 42 in immunocompetent participants (Table 3). Among the 54 episodes of laboratory confirmed COVID-19 in IC, three (5.6%) occurred less than 14 days after the first vaccine dose, 36 (66.7%) after the third dose and 11 (20.4%) after the fourth dose. Among the 42 episodes in immunocompetent participants, 29 (69%) occurred after the third dose. Of the 96 (44.8%) COVID-19 episodes, 43 occurred from January 3^rd^ to February 17^th^, 2022, during the first Omicron wave in Brazil. Three asymptomatic IC participants had COVID-19 confirmed by PCR.

**Table 3:** COVID-19 episodes in immunocompromised (IC) and immunocompetent (H) participants during the study period.

Variables	Immunocompromised (N=54)		Immunocompetent (N=42)	
	
	N	%	n	%
Time of the COVID-19 episode*				
<14 days after the 1^st^ vaccine dose	3	5.6	0	0.0
Post-V1	1	1.9	0	0.0
Post-V2	3	5.6	13	31.0
Post-V3	36	66.7	29	69.0
Post-V4	11	20.4	-	-
**Symptomatic COVID-19**	**51**	94.4	42	100.0
**Healthcare use**	38/51	70.4	40	95.2
**Hospitalization**	**13**/51	24.1	0	0.0
ICU	8/13	61.5	-	-
Intubation / mechanical ventilation	6/7	85.7	-	-
Non-invasive ventilation or high-flow catheter	6/7	85.7	-	-
Nasal catheter or mask	7/7	100.0	-	-
**Oxygen**	**7**/13	53.8	-	-
Vasoactive drugs	6/13	46.2	-	-
Dialysis	2/13	15.4	-	-
ECMO	1/13	7.7	-	-
**COVID-19 outcome**				
Ongoing	1	1.9	0	0.0
Recovered	45	83.3	41	97.6
Recovered with sequelae	3	5.6	1	2.4
Not recovered	1	1.9	0	0.0
Death	4	7.4	0	0.0
**Variant**				
P.1 SARS-COV-2	3	5.6	0	0.0
20J (GAMMA, V3) (P.1)	1	1.9	1	2.4
21J (DELTA) (AY.99.2)	0	0.0	2	4.8
21A (DELTA) (B.1.617.2)	1	1.9	3	7.1
21K (OMICRON)	2	3.7	1	2.4
21K (OMICRON) - (BA.1)	11	20.4	3	7.1
21K (OMICRON) - (BA.1.1)	3	5.6	4	9.5
21K (OMICRON) - (BA.1.1.1)	0	0.0	1	2.4
22A (OMICRON) - (BA.4)	2	3.7	5	11.9
22B (OMICRON) - (BA.5.2.1)	1	1.9	2	4.8
21L (OMICRON) - (BA.2)	4	7.4	2	4.8
21L (OMICRON) - (BA.2.47)	0	0.0	2	4.8
Other	3	5.6	9	21.4
Inconclusive	10	18.5	3	7.1
Not informed	13	24.1	4	9.5

*PCR or antigen test positive ≥15 days after dose administration was considered post-vaccine dose.

Among IC participants with symptomatic COVID, 13 out of the 54 (24.1%) needed hospitalization and of these, eight (61.5%) were admitted into Intensive Care Unit, whereas no H participants needed hospitalization. Four of the 54 (7.4%) IC participants died and 45 (83.3%) recovered without sequelae, whereas 41 of the 42 (97.6%) H participants recovered without sequelae and none died.

Among the identified SARS-CoV-2 variants, Omicron was the most frequent in both groups (40/66 or 60.6%), of which 14 (35%) were BA.1.

## DISCUSSION

In the present study, a 2-dose CoronaVac schedule led to statistically significant lower anti-N, anti-S and anti-RBD SCR, GMT and GMTR levels in immunocompromised individuals compared with immunocompetent (healthy) adults. A third dose of mRNA BNT162b2 vaccine increased anti-S and anti-RBD GMT and SCR in IC, but SCR values were still lower in IC than in healthy participants with two CoronaVac doses. After the 3^rd^ BNT162b2 dose, IC had lower anti-RBD GMT but higher anti-S GMT than H after the 2^nd^ CoronaVac dose. As expected, the third dose had no effect on anti-N antibody, since mRNA vaccines contain only S-antigen^12^. A BNT162b2 booster (4^th^ dose for IC and 3^rd^ dose for H) led to an increase in anti-S and anti-RBD GMT in both groups, but IC still presented lower GMT and lower SCR. These findings strengthen the need for a 3-dose primary COVID-19 vaccination schedule for immunocompromised individuals and the high immunogenicity of mRNA vaccine heterologous booster after immunization with an inactivated vaccine in this population. We also observed an increase in anti-N SCR (from 51.9% after the 2^nd^ CoronaVac dose to 75.3% at trial end) and GMT (from 2.3 after the 2^nd^ CoronaVac dose to 10.6 at trial end) in the IC group, and from 15.1 to 33.6, respectively, in H, despite BNT162b2 not eliciting any response to anti-nucleocapsid. This increase probably resulted from natural infection by COVID-19 that was still circulating in Brazil. The 96 COVID-19 episodes confirmed by a positive SARS-CoV-2 PCR or antigen test diagnosed during the study strengthen this hypothesis.

Our results agree with previous studies that reported lower immunogenicity of the 2-dose CoronaVac schedule in a cohort of persons living with HIV (PLH)^10^, individuals with immune-mediated^8^ and rheumatic diseases^13^, kidney transplant recipients^14^, solid organ cancer patients receiving active treatment^15^, and in a cohort of subjects with various immunocompromising conditions^16^. Other investigations have reported lower immunogenicity of other COVID-19 vaccines^17^ in IC, such as SOT^18^, rheumatic immune-mediated diseases^19^, glomerular diseases^17^ and IEI^20^. These findings repeat previous experience with other vaccines.

An increase in seroconversion and GMT after heterologous mRNA vaccine booster following a 2-dose CoronaVac schedule was previously reported in SOT recipients^21^ and cancer patients^15^. Six months after the 2^nd^ dose, a homologous third CoronaVac dose also resulted in enhanced humoral response in rheumatic patients: anti-S1/S2 IgG seropositivity increased from 60% (pre-) to 93% (post-booster) and neutralizing antibody positivity increased from 38% (pre-) to 81.4% (post-booster)^22^.

Studies have also demonstrated the importance of an additional in immunocompromised individuals that received other vaccines^23^. Among patients with lymphoid malignancies, 53.7% seroconverted after two doses of mRNA vaccine and a third dose increased SC to 68.8%^24^. Among those who seroconverted after the 2^nd^ dose, the third dose enhanced anti-S IgG to titers similar to those observed in healthy adults^24^. Among cancer patients who were anti-S seronegative 4-6 months after the primary vaccination schedule, 56% seroconverted after a third dose^25^. In a cohort of 96 heart transplant recipients, administering a third BNT162b2 dose 168 days after the 2^nd^ dose increased seropositivity from 23% to 67%^16^. A 9-fold increase in SARS-CoV-2 neutralization titers and 3-fold increase in IgG anti-RBD antibodies were observed after the third dose^16^.

Although we did not evaluate the immune responses of subgroups with different immunocompromising conditions, other studies on COVID-19 vaccines immunogenicity found better immune responses in PLH, possibly related to antiretroviral therapy, and immune-mediated inflammatory diseases, maybe due to the lower levels of immunosuppression they are submitted to compared with SOT recipients and IEI who present lower responses^10,18,20^.

Older age (≥60 years) immune response to COVID vaccines differs from IC. Older adults had values closer to the healthy/general population than IC results^6,8,21,26^. Immunosuppressive drugs may also play a role: anti-CD20 (rituximab) and mycophenolate mofetil (MMF) were associated with lower immune response in several studies^6,15,23-26^. Bruton Tyrosine Kinase inhibitors (BTKi) were associated with lower immune response in chronic lymphocytic leukaemia^27^. Prednisone (≥20mg daily), methotrexate, TNF-inhibitors, MMF, rituximab and abatacept were all associated with lower seroconversion in individuals with rheumatic diseases^11^. In HSCT recipients, vaccination ≥6 months after HSCT was associated with better immune response^28^.

Few studies compared different types of COVID-19 vaccines in immunocompromised subjects. One study found that a 2-dose schedule of CoronaVac or mRNA/Pfizer led to a similar seroconversion (95.7% vs. 100%) as in the healthy population^29^. However, SCR values were lower after CoronaVac (78.7%) but not mRNA (100%) and IgG and neutralization titers were observed in adults with rheumatic diseases^29^. Another study evaluated different 2-dose vaccination schedules in persons with rheumatic diseases and found that inactivated vaccines resulted in the lowest immune response whereas heterologous AZD122+BNT162b2 led to the highest antibody titers^30^.

In kidney transplant recipients who received the 2-dose CoronaVac schedule, heterologous mRNA BNT162b2 vaccine booster resulted in higher seroconversion (49%), higher seropositivity (67%) and higher anti-RBD titers than a homologous booster (32% of seroconversion and 55% of seropositivity)^23^. A Chilean study involving SOT patients found that heterologous mRNA BNT162b2 vaccine booster after the 2-dose CoronaVac schedule resulted in lower neutralizing antibody positivity (55.1%) than three doses of mRNA vaccine (77.4%)^20^. This study also evaluated different vaccines in different immunocompromising conditions and found that mRNA-1273 vaccine was associated with overall significantly higher anti-RBD titers (mean 10.24) than mRNA BNT162b2 (5.25) and adenovirus vector vaccines (1.82)^20^.

Immunogenicity results must be interpreted with caution, since immune correlates of protection for SARS-CoV-2 are unknown. However, real-world studies have reported reduced COVID-19 vaccines effectiveness in the immunocompromised compared with the healthy/general population, most of them conducted in high-income countries that mainly used mRNA or vector-based vaccines^30^. Between different immunocompromising conditions, better effectiveness has been reported in immune-mediated rheumatic patients and the lowest effectiveness in SOT recipients, in line with immunogenicity data^31^.

This study has limitations. First, different immunocompromising conditions and immunosuppressive therapy as well as different individual genetic background may result in different immune responses to vaccines. Our small sample size did not allow for subgroups statistical analysis. Second, we were unable to compare mRNA BNT162b2 vaccine to other heterologous or homologous boosters. Third, data on time interval between chemotherapy, transplant, and use of immunosuppressive drugs and vaccination were unavailable. Fourth, cellular immunity was not assessed. Fifth, our assay results were quite different compared with those in the literature posing a difficulty to comparison with other studies. Finally, the presented data are the result of both vaccination and infection, since SARS-CoV-2 virus transmission was high throughout the study period and many COVID-19 cases were diagnosed among participants during the study.

Despite these limitations, our study included participants with severe immunocompromising conditions and demonstrated the immunogenicity of two CoronaVac doses plus two additional mRNA vaccine boosters with results similar to other heterologous schedules, such as viral vector vaccine followed by mRNA vaccine^32^.

## CONCLUSION

This prospective study demonstrated that CoronaVac had acceptable short-term immunogenicity in individuals with different immunocompromising conditions (SOT, HSCT, IEI, cancer and rheumatic patients). BNT162b2 heterologous booster enhanced immune response in IC. However, IC had lower humoral immune responses than immunocompetent participants, even after two additional doses.

The third pandemic year was marked by significantly reduced hospitalizations and deaths, mainly due to increased population immunity related to both vaccination and infection (hybrid immunity). The virus continued evolving with new variants emerging but, so far, it has stabilized. In this scenario, immunocompromised individuals continue to be at greater risk of severe disease and death due to lower vaccine immunogenicity/effectiveness or more rapid waning immunity in this population group. Periodic boosters (every 6–12 months), probably with updated vaccines, remain necessary to protect more vulnerable individuals^33^.

Improving vaccine immunogenicity in the immunocompromised requires strategies such as pausing immunosuppressive therapy for vaccination, and revaccination or boosting after discontinuing immunosuppression. An appropriate vaccine schedule should always consider differences between different immunocompromising conditions/therapies to improve response and support trust in vaccination.
