# Erratum

**DOI:** 10.1590/S1678-9946202567024err

**Published:** 2025-04-04

On the article "Immunogenicity of COVID-19 adsorbed inactivated vaccine (CoronaVac) and additional doses of mRNA BNT162b2 vaccine in immunocompromised adults compared with immunocompetent persons", published in the Revista do Instituto de Medicina Tropical de São Paulo, Vol. 66, e24, p. 2024 (http://doi.org/10.1590/S1678-9946202466024):

Where it reads Maria Isabel de Moraes Pinto^6^ should be read Maria Isabel de Moraes Pinto^11^

